# A Common Garden Test of Host-Symbiont Specificity Supports a Dominant Role for Soil Type in Determining AMF Assemblage Structure in *Collinsia sparsiflora*

**DOI:** 10.1371/journal.pone.0055507

**Published:** 2013-02-05

**Authors:** Shannon P. Schechter, Thomas D. Bruns

**Affiliations:** Department of Plant and Microbial Biology, University of California, Berkeley, California, United States of America; University of California Riverside, United States of America

## Abstract

Specialization in plant host-symbiont-soil interactions may help mediate plant adaptation to edaphic stress. Our previous field study showed ecological evidence for host-symbiont specificity between serpentine and non-serpentine adapted ecotypes of *Collinsia sparsiflora* and arbuscular mycorrrhizal fungi (**AMF**). To test for adapted plant ecotype-AMF specificity between *C. sparsiflora* ecotypes and field AMF taxa, we conducted an AMF common garden greenhouse experiment. We grew *C. sparsiflora* ecotypes individually in a common pool of serpentine and non-serpentine AMF then identified the root AMF by amplifying rDNA, cloning, and sequencing and compared common garden AMF associates to serpentine and non-serpentine AMF controls. Mixing of serpentine and non-serpentine AMF soil inoculum resulted in an intermediate soil classified as non-serpentine soil type. Within this common garden both host ecotypes associated with AMF assemblages that resembled those seen in a non-serpentine soil. ANOSIM analysis and MDS ordination showed that common garden AMF assemblages differed significantly from those in the serpentine-only controls (R = 0.643, *P*<0.001), but were similar the non-serpentine-only control AMF assemblages (R = 0.081, *P*<0.31). There was no evidence of adapted host ecotype-AMF specificity. Instead soil type accounted for most of the variation AM fungi association patterns, and some differences between field and greenhouse behavior of individual AM fungi were found.

## Introduction

Plant adaptation to complex soil factors is not fully explained by plant physiological and morphological traits [1], [2]. Specialization in plant host-symbiont-soil interactions may be an important mechanism to mediate plant adaptation to environmental stress [3]–[5]. Theory predicts that low quality environments favors the evolution of specificity in host-mutualist interactions [3] and this co-evolution could confer adaptive tolerances to environmental stress [5]. However, host-symbiont specificity has yet to be shown as a direct mechanism for plant adaptation to environmental stress.

Serpentine soils are well known edaphic (soil physical, chemical, and biological characteristics) extremes that create locally adapted plant ecotypes [6]. Although the process of plant adaptation to serpentine has been studied for several decades, the mechanisms of plant adaptation to serpentine are still poorly understood [2], [7], [8]. Approximately 85% of all plants interact with the soil environment through symbiosis with arbuscular mycorrhizal fungi (**AMF**) [9]. These common root symbionts (Glomeromycota) have co-evolved with plants for over 400 million years [10] and are known to increase the plant hosts' establishment and growth in stressful environments [11]. Therefore, symbiosis with AMF may be a key evolutionary strategy for plant adaptation to edaphic stress.

Thrall et al. [4] suggested three mechanisms that would generate evolutionary shifts in host plant-soil symbiont interactions that could mediate plant adaptation to stress. First is a symbiont-driven adaptation to stress in which plant success depends on association with stress-adapted symbiont while host dependence on the symbiosis is unchanged across the stress gradient. An example of this is arsenate resistant AM fungus *Glomus mosseae* conferring resistance to it non-adapted host *Holcus lanatus* under arsenic stress [12]. The second proposed mechanism is change in host dependence (increased or decreased) on the soil symbionts along an environmental gradient [4]. An example of this is the high-nutrient adapted ecotype of *Andropogon gerardii* evolving reduced dependence on the AM symbiosis [13]. The third mechanism is co-adaptation of host and symbiont requiring genetic changes in both partners for adaptation and persistence under environmental stress [4]. In this case, host-symbiont specificity plays a large role resulting in adapted plant genotypes doing best with adapted symbiont genotypes.

Host-symbiont specificity between stress adapted plant host and adapted AM fungi has yet to be shown. However, recent studies have laid the empirical foundation for this process. Kiers et al. [14] has shown that plants can detect, discriminate, and preferentially reward the most cooperative AM fungus and that individual AM fungi can discriminate among hosts differing in C-supply. This verifies work by Bever et al. [15] which showed preferential allocation of fixed carbon to the more beneficial AM fungal symbiont. In addition, two molecular genetic studies have shown that individual genotypes of the AM fungus *Glomus intraradices* vary in host plant preference [16] and mycorrhizal symbiotic effectiveness [17]. Finally, Helgason *et al.* [18] found host-symbiont “selectivity” between host *Acer pseudoplantanus* and its AM fungal symbiont *Glomus hoi* across soil types, growth conditions, and P nutrient levels. However, no one has tested host-symbiont specificity between stress adapted host genotypes and AM fungi.

There is ecological evidence for host-symbiont specificity between serpentine adapted plant ecotypes and AM fungi. We characterized and compared the AMF assemblages associated with experimentally determined serpentine and non-serpentine adapted ecotypes of *Collinsia sparsiflora* from six sites within close geographical range (110 m to 1.94 km between sites) [19]. We found that serpentine and non-serpentine *C. sparsiflora* ecotypes associated with distinct AMF assemblages: *Acaulospora* sp.1-dominated serpentine, and *Glomus* sp. 1-dominated non-serpentine ecotype assemblage along with other less abundant AMF taxa that also showed a potential plant ecotype bias. However, we also found a relationship between plant ecotype AMF assemblages and rhizosphere soil nutrient status [19], thus opening up the possibility that soil or host or both factors could be responsible for the distinction between plant ecotype AMF assemblages. Since we did not see evidence of AMF dispersal limitation between *C. sparsiflora* ecotype locations, we suggested two scenarios that might explain the distinction between plant ecotype AMF assemblages: 1) specificity between adapted plant genotypes and adapted AM fungal genotypes within a ubiquitous AMF assemblage 2) nonspecific association between plant ecotypes and AMF assemblages that had been shaped by edaphic factors [19]. This study tests the first scenario as an AMF common garden experiment.

The goal of this study is to determine if the distinction between AMF assemblages associated with serpentine and non-serpentine *C. sparsiflora* ecotypes in the field [19] was due specificity between adapted plant genotypes and adapted AM fungi genotypes within a ubiquitous AMF assemblage. To do this, we conducted an AMF common garden experiment in which *C. sparsiflora* serpentine and non-serpentine ecotypes were grown individually with a common pool of serpentine and non-serpentine AMF under greenhouse conditions. In order to determine if the plant ecotypes select specific AMF taxa from the common garden, we identified the root AMF associates of each plant ecotype via molecular methods. We hypothesized that specificity between *C. sparsiflora* ecotypes (i.e. genotypes) and AM fungal genotypes would be indicated by a similar pattern of associated taxa when grown in a common AMF pool as found in the field. Specifically, we expected *Acaulospora sp.* 1 to be the dominant AMF associate in the *C. sparsiflora* serpentine ecotypes and *Glomus sp.* 1 to be dominant in the non-serpentine ecotypes independent of edaphic conditions. Alternatively, finding random associations between the plant ecotypes and AMF taxa within a common AMF pool would indicate that no specificity exists between adapted host ecotypes.

## Materials and Methods

### Study System

Seeds, soil, and AMF for this study were collected at the Donald and Sylvia McLaughlin University of California Natural Reserve situated in Napa, Lake, and Yolo counties in northern California. We collected from the same serpentine and non-serpentine *Collinsia sparsiflora* ecotype populations as described in Schechter and Bruns [19]. In summary, *C. sparsiflora* (Plantaginaceae) is a small California native annual that germinates, flowers, sets seed and then dies, the timing of this annual cycle is defined by seasonal rains and under normal field conditions is completed within a three to four month period [20]. Serpentine and non-serpentine ecotypes of *C. sparsiflora* were previously demonstrated through a reciprocal transplant experiment [20].

### Soil Collection

In March 2006, we collected field rhizosphere soil and roots of *C. sparsiflora* from four populations: two serpentine (S1 and S2) and two non-serpentine (NS1 and NS3) [19]. These four field collections served as the sources of AMF inoculum for the common garden. We chose these four *C. sparsiflora* ecotype populations because they were the best representatives of serpentine and non-serpentine ecotype populations and AMF from our previous study [19]. We collected the *C. sparsiflora* rhizosphere soil and roots in the field adjacent to the same patches sampled by Schechter and Bruns [19]. We equalized the number of plants sampled from each site. We removed all above-ground plant material, equalized the volume of roots per sample and cut the roots into 1 cm segments. We then dried each collection for 24 hours in the fume hood at room temperature to limit microbe activity and ease mixing. The dried collections were stored at 4°C up to one week until used for planting.

### Experimental Design

In order to test if *C. sparsiflora* ecotypes selected specific AMF from a “common garden” of serpentine and non-serpentine AMF, we grew individual seedlings of each *C. sparsiflora* ecotype in separate pots in a mix of serpentine and non-serpentine AMF soil inoculum. The common garden of serpentine and non-serpentine AMF was produced by thoroughly mixing equal amounts of the field collected *C. sparsiflora* rhizosphere soil and roots (S1, S2, NS1, and NS3) and then diluting this mixture 1∶1 (volume to volume) with sterilized sand to facilitate drainage.

### Planting

We collected *C. spariflora* seeds from the four field populations (S1, S2, NS1, and NS3) in May 2005. These seeds were pregerminated in 1% water agar and transplanted into individual “stubby cell” cone-tainers (6.5 cu. in. Stuewe and Sons) filled with the soil∶sand (AMF common garden) mixture. Twenty replicates of each ecotype population were transplanted for a total of 80 seedlings for the experiment. Seedlings were arranged in a completely randomized design and grown in a greenhouse (UC Berkeley, Berkeley, CA, 37°52′29″N 122°16′2″W) from April until flowering in June 2006 (maximum temperature: 74°F, minimum temperature: 60°F). The seedlings were sub-irrigated by placing the cone-tainer trays in a tub of water as needed. Sub-irrigation is the best watering method for this plant species (J. Wright, *pers. comm.*) No fertilizer was used in the experiment.

Controls- serpentine and non-serpentine AMF controls were also planted for this experiment. We knew that sampling, processing, and greenhouse growth conditions could affect the AMF assemblages associating with *C. sparsiflora* ecotypes, so the serpentine and non-serpentine AMF soil controls were used to assess these factors and individual soil type affects on AMF assemblages as a baseline for comparison. For the serpentine-only AMF control, we transplanted four replicate seedlings from the four *C. sparsiflora* ecotype populations (S1, S2, NS1, and NS3) for a total of 16 grown in an equal mix of rhizosphere soil and roots from S1 and S2 *C. sparsiflora* serpentine populations combined 1∶1 with sterile sand. For the non-serpentine-only AMF control, we transplanted four replicate seedlings from the four *C. sparsiflora* ecotype populations (S1, S2, NS1, and NS3) for a total of 16 grown in an equal mix of rhizosphere soil and roots from NS1 and NS3 *C. sparsiflora* serpentine populations combined 1∶1 with sterile sand. Roots of the controls were harvested and AMF associates identified in the same manner as the common garden experiment seedlings (see below). However, S1 plants growing in non-serpentine-only control grew poorly and resulted in insufficient root tissue mass for downstream molecular identification of AMF associates. We did not measure any above-ground parameters for these controls.

### Harvest

All seedlings were harvested after flowering since these annual plants senesce soon after completion of flowering (*pers. obs.*). However, this harvest process was completed for all plants within a two week time period. Only ten of the twenty seedlings from the S2 population survived to flowering stage for reasons unknown. We measured plant height, the number of flowers, shoot and root dry weight, AMF colonization, and identified root associated AMF taxa via molecular methods (see below). We sampled soil from ten randomly selected harvested seedlings and pooled five together for two common garden soil samples sent to A & L Western Agricultural Laboratories for chemical analysis. We dried the shoots in a 37°C oven for three days before weighing them. Roots of individual seedlings were thoroughly washed to remove as much soil as possible. We took a small portion (5 mg wet weight) of the washed roots to quantify AMF colonization using a compound microscope [21], and dried the rest of the root tissue in a 37°C oven for three days. The dried roots were weighed and then stored in a −80°C freezer until DNA extraction.

### Molecular Analysis

#### DNA extraction

We extracted DNA from each *C. sparsiflora* root sample as previous described Schechter and Bruns [19]. Briefly, we crushed the dried and frozen roots by beadbeating (Mini-Beadbeater, Biospec Products) with sterile glass beads and 1.5 ml of 2× CTAB buffer (2% CTAB, 1% PVP, 0.1 M Tris pH 8.0, 1.4 M NaCl, 0.02 M EDTA) was added to the cryotube. We used a chloroform∶isoamyl alcohol extraction method to extract DNA from these samples, and extracts were purified using the DNeasy Tissue Kit (Qiagen).

#### Polymerase chain reaction (PCR)

PCR reactions and conditions were the same as described by Schechter and Bruns [19]. Controls- each replicate of the control *C. sparsiflora* root samples was amplified using PCR under conditions described below, for a total of 32 PCR reactions. Common Garden- due to the fact that only ten seedlings of the S2 population survived to harvest, we equalized the number of seedlings analyzed for AMF associates by randomly choosing ten root-DNA extracts from each of the other *C. sparsiflora* ecotype populations for PCR amplification. This resulted in a total of 40 PCR reactions (ten from each ecotype population). We amplified a variable region of the 18S rDNA using *Pfu Turbo* DNA polymerase (Stratagene) and universal eukaryotic primer NS31 [22] paired with AM1 [23].

#### Cloning and Sequencing

Pooling PCR of replicate samples prior to cloning and sequencing has been shown to detect similar levels of AMF diversity as cloning and sequencing individual PCR reactions from single replicates [24]. Therefore we pooled PCR of replicate samples prior to cloning and sequencing to reduce cost and time without sacrificing diversity. Controls- we pooled PCR products from all four replicates of each ecotype population-soil type treatment combination for cloning under the conditions described below. Common Garden- we pooled the PCR products from two replicates of the same ecotype together for cloning to equal five cloning reactions per ecotype population. We first gel purified and concentrated the pooled PCR products before cloning as described by Schechter and Bruns [19]. We then cloned the pooled PCR products into pPCR-Script Amp SK(+) and transformed into *Escherichia coli* XL10-Gold Kan Ultracompetent cells (Stratagene). We screened transformants for correctly sized inserts using plasmid primers T3/T7 under the same PCR conditions as described by Schechter and Bruns [19]. Then, we selected gel confirmed positive transformants for cleaning and sequencing. We cleaned these PCR products with ExoSAP-IT using the manufacturer's instructions (USB), and sent the clean PCR products to the UC Berkeley Sequencing Facility (Berkeley, CA) for sequencing in one direction with AM1. We edited the sequences using Sequencher 4.2.2 (Gene Codes) and eliminated vector sequences using VecScreen (http://www.ncbi.nlm.nih.gov/VecScreen/). Representative AMF sequences were deposited into GenBank (**HQ342700**–**HQ342752**). Chimeras were detected as described by Schechter and Bruns [19] and suspect sequences eliminated from the data set.

### Data Analysis

#### Operational Taxonomic Unit (OTU) determination

We determined AMF OTUs in this experiment using the same combination of sequence similarity and phylogenetic analysis methods described by Schechter and Bruns [19]. Consistency in OTU determination methods allows us to compare OTUs between studies as required to test our hypotheses. We first combined sequences obtained from each cloning reaction (pooled PCR products from two seedling replicates of the same ecotype population) at 98% similarity using Sequencher 4.2.2 to create AMF sequence contigs and singletons associated with each pair of seedling replicates (referred to hereafter as “paired-seedlings”). Then we compared all contigs and singletons together at 98% to determine sequence similarity groupings for the entire data set; these groupings were used to define OTUs.

We aligned these sequences along with those sequences used in Schechter and Bruns [19] using ClustalX [25] and then manually edited the alignment using MacClade v 4.08 [26]. Two separate phylogenetic analyses were performed using *Olpidium brassica* as an outgroup: maximum likelihood (ML) analysis was conducted using Garli (Genetic Algorithm for Rapid Likelihood Inference) v 0.95 [27], and Bayesian analysis was performed using MrBayes 3.1.1 [28]. These analyses were conducted using the same methods described by Schechter and Bruns [19].

We used the results of the phylogenetic analyses to confirm OTUs as in Schechter and Bruns [19]. This included looking for consistency in topology between analyses and >50% bootstrap or Bayesian posterior probability branch support for clades that included the putative OTU sequences (98% sequence similarity groupings). These OTUs were then used to determine the assemblages of AM fungi associated with each of the *C. sparsiflora* paired-seedlings.

#### Assemblage Analyses

AMF assemblages were analyzed in the same manner described by Schechter and Bruns [19]. We used the PRIMER 5 software (Plymouth Routines in Multivariate Ecological Research) [29] to perform the AMF assemblage analyses. We prepared a relative abundance matrix of OTUs present in each of the paired-seedlings root samples based on the number of clones representing those OTUs. We then produced a similarity matrix using the Bray-Curtis similarity measure after performing a square-root transformation. We used non-metric Multidimensional Scaling (**MDS**) ordinations to represent the dissimilarities in assemblage composition among samples and the **ANOSIM** (analysis of similarities) routine to perform statistical analysis of assemblage data [29].

We produced a rarefaction curve to determine if clone sampling effort saturated the number of OTUs using the EstimateS 8.0 Mao Tau estimator [30]. We also used PRIMER 5 to compute Shannon-Wiener diversity (H′), richness, and evenness for each ecotype population, and tested for differences between ecotype populations in the univariate indices using one-way ANOVA (JMP v. 5). Tukey HSD tests were used for all *a posteriori* comparison of means.

#### Plant Harvest

We used one-way ANOVA (JMP v. 5) to test for differences between ecotype populations in the plant height, number of flowers, root and shoot dry weight, and colonization (arcsine transformed). Tukey HSD tests were used for all *a posteriori* comparison of means.

#### Post-hoc Analysis (Field vs. Common Garden)

Analysis of the common garden experiment led to a decision to look back at data presented by Schechter and Bruns [19] as a means to understand the results. We used the PRIMER 5 software to compare differences in soil chemical characteristics between common garden soil and field soil collected from the S1, S2, NS1 and NS3 ecotype population sites [19]. Soil chemical data was log transformed, and then the similarity matrix was produced using Euclidean distance [29]. Non-metric MDS was used to demonstrate differences in soil chemical characteristics between samples. One-way ANOVA (JMP v. 5) was also used to compare soil chemical data (log transformed) between common garden experiment soil and field soil collected from the S1, S2, NS1 and NS3 ecotype population sites [19]. Tukey HSD tests were used for all *a posteriori* comparison of means.

## Results

### Plant Harvest Data

There were no significant differences between *C. sparsiflora* ecotype populations grown in the common garden soil in any growth parameter (Table 1). Plant height (F_3,39_ = 0.89, *P*<0.89), number of flowers (F_3,39_ = 1.18, *P*<0.34), shoot dry weight (F_3,39_ = 0.51, *P*<0.68), root dry weight (F_3,39_ = 0.45, *P*<0.72), shoot+root dry weight (F_3,39_ = 0.42, P<0.74), and AMF colonization (F_3,39_ = 0.13, P<0.94) were all similar across ecotype populations.

**Table 1:** Results of the harvest of *Collinsia sparsiflora* ecotype populations (S1, S2, NS1, and NS3) after being grown in a common garden of serpentine and non-serpentine AM fungi.

Ecotype	Height (cm)	Shoot dry weight (g)	Root dry weight (g)	Shoot+Root dry weight (g)	Colonization (%)
S1	14.15 **(2.43)**	0.10 **(0.03)**	0.016 **(0.007)**	0.12 **(0.04)**	55.50 **(9.89)**
S2	13.80 **(1.78)**	0.09 **(0.03)**	0.015 **(0.004)**	0.10 **(0.03)**	52.00 **(11.51)**
NS1	12.85 **(1.95)**	0.11**(0.04)**	0.019 **(0.010)**	0.13 **(0.04)**	54.20 **(5.33)**
NS3	14.82 **(1.50)**	0.11**(0.04)**	0.014 **(0.005)**	0.13 **(0.04)**	52.60 **(9.97)**

Values are means (N = 10) with standard deviation in parentheses. No significant differences (*P*<0.05) were found for any parameter measured.

### Assemblage Identification

#### Common Garden

A total of 1,543 clones were sequenced (96% AMF sequences, 1.2% bacterial origin, 0.4% ascomycota origin, 0.1% plant origin, and 0.4% chimeric sequences). Each *C. sparsiflora* ecotype population was represented by similar numbers of AMF sequences (S1 = 340, S2 = 427, NS1 = 367, NS3 = 367). We detected only three AMF genera in this study (Fig. S1, *if accepted*). *Glomus* species were the most abundant by far, representing 99.8% of the AMF sequences. The two other genera are both in the Archaeosporales: *Archaeospora* (0.1%), and a newly described genus *Ambispora* (0.1%) [31]. Using the combined sequence similarity and phylogenetic criteria for OTU determination, we established 10 AMF OTU (Table 2), seven of which matched OTUs (at 98% sequence similarity, see Figure S1) found in Schechter and Bruns [19] (Fig. S1). Of these, two OTU were the most abundant sequence types: *Glomus sp.* 1 (47%) and *Glomus* 4 (20%). *Glomus sp.* 1 was the most dominant AMF OTU across ecotype populations, having the highest average relative abundance in each population.

**Table 2:** Relative abundance matrix of AM fungal taxa associated with serpentine and non-serpentine ecotypes of *Collinsia sparsiflora* growing in a common garden of serpentine and non-serpentine AM fungi.

	GROWTH MEDIA										
	Serpentine Only				Common Garden				Non-serpentine Only		
OTU^a^	S1^b^	S2^b^	NS1^c^	NS3^c^	S1	S2	NS1	NS3	S2	NS1	NS3
***Glo 1***	*4*	*0*	*0*	*8*	*36*	*68*	*46*	*31*	*36*	*31*	*42*
Glo 2	12	0	8	0	9	0	1	0	0	0	0
***Glo 4***	*42*	*41*	*73*	*54*	*21*	*11*	*24*	*27*	*14*	*6*	*25*
Glo 5	0	0	0	0	3	4	6	13	23	19	0
Glo 6	8	5	15	15	6	4	4	2	18	0	0
**Glo 7**	*12*	*27*	*4*	*8*	*17*	*9*	*4*	*6*	*5*	*25*	*0*
Glo 8	4	0	0	0	0	0	0	0	0	0	0
***Glo 9***	*15*	*23*	*0*	*8*	*7*	*4*	*18*	*21*	*5*	*19*	*33*
Glo A	0	0	0	0	0	0	0	1	0	0	0
Glo B	0	5	0	0	0	0	0	0	0	0	0
Acaul 1	0	0	0	8	0	0	0	0	0	0	0
Amb 1	0	0	0	0	1	0	0	0	0	0	0
Arch 2	2	0	0	0	0	0	1	0	0	0	0
Scut 1	4	0	0	0	0	0	0	0	0	0	0

Note: S1 plants growing in non-serpentine-only control grew poorly and resulted in insufficient root tissue mass for downstream molecular identification of AMF associates. Italicized OTUs show ecotype effects. Bold type OTUs are discussed in text.

a Operational taxonomic unit,

b serpentine ecotype populations,

c non-serpentine ecotype populations.

#### Controls

AMF assemblages of the *C. sparsiflora* ecotypes populations growing in the serpentine-only control were different from those growing in the non-serpentine-only control (Table 2). AMF assemblages of ecotype populations growing in the serpentine-only control were dominated by taxa in the genus *Glomus* (98% of sequences) especially by the *Glomus* 4 OTU (53%), but also included sequences belonging to the genera *Acaulospora* (*Acaul* 1, 1%) and *Scutellospora* (*Scut* 1, 1%). In contrast, AMF assemblages of the ecotype populations growing in the non-serpentine-only control contained only *Glomus* taxa sequences, and were dominated by sequences of the *Glomus sp.* 1 OTU (36%). All OTUs but one (*Glomus* C) matched the OTUs found in Schechter and Bruns [19] (Fig. S1).

### *Assemblage Analysis*

#### Comparing assemblages

The rarefaction analysis indicated that the sequence sampling effort was sufficient for a comparison of AMF assemblages associated with each ecotype population (Fig. S2). AMF composition was similar across ecotype populations growing in the common garden soil. ANOSIM analysis of presence/absence of AMF OTUs showed no differences in AMF composition between ecotype populations (R = 0.026, *P* = 0.196). ANOSIM analysis based on relative abundance data, showed that the S1 and S2 associated AMF assemblages were both different from the NS1 ecotype population (S1: R = 0.32, *P* = 0.04; S2: R = 0.46, *P* = 0.03) but neither was different from the NS3 ecotype population AMF assemblage (S1: R = 0.16, *P* = 0.15; S2: R = −0.04, *P* = 0.65). In fact, the S1 and S2 ecotype population AMF assemblages were different from each other (R = 0.30, *P* = 0.03), while the NS1 and NS3 ecotype populations had similar AMF assemblages (R = 0.2, *P* = 0.09). Similarity percentages analysis indicated that variation in relative abundances of *Glomus sp.* 1, *Glomus*, 4, *Glomus* 7, and *Glomus* 9 OTUs drove these differences between ecotype populations.

**Figure 1:**
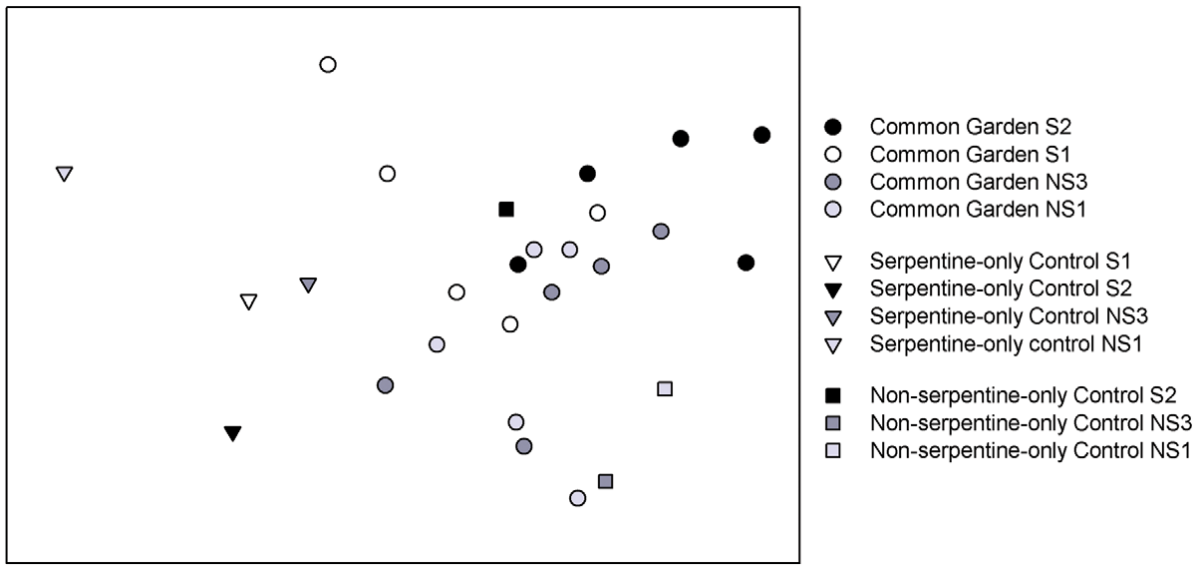
Non-metric multi-dimensional scaling ordination of AMF assemblages associated with ecotype populations (S1, S2, NS1, and NS3) of *Collinsia sparsiflora* grown in a common garden of serpentine and non-serpentine AMF (each point represents AMF assemblage from a pooled sample of two seedling replicates), serpentine soil control or non-serpentine soil control AMF (each data point represents AMF assemblage from a pooled sample of four seedling replicates). The non-metric multi-dimensional scaling ordination is a configuration of the samples in which relative positions are assigned based on the Bray–Curtis similarity matrix of the data so that samples closer together have a higher similarity of component taxa than samples farther apart and overlapping samples are highly similar. The nonmetric scale of the ordination does not assign values to the axes. Note: S1 plants growing in non-serpentine-only control grew poorly and resulted in insufficient root tissue mass for downstream molecular identification of AMF associates.

When comparing AMF assemblages found in the common garden with those found in the serpentine-only and non-serpentine-only controls, it is obvious that the common garden AMF assemblages are much more similar to those found in the non-serpentine-only controls than the AMF assemblages of the serpentine-only control (Figure 1). The ANOSIM analysis confirmed this with the common garden AMF assemblages significantly different from those in the serpentine-only controls (R = 0.643, *P*<0.001), but not different from the non-serpentine-only control AMF assemblages (R = 0.081, *P*<0.31). The serpentine-only control AMF assemblages were significantly different from those of the non-serpentine-only controls (R = 0.944, P<0.03), but no differences were detected between the ecotype populations (R = −0.704, *P*<0.97). Taken together, these data show that the two *C. sparsiflora* ecotypes behave similarly with respect to AMF and both associate with distinct soil-defined AMF assemblages that in the common garden setting resemble that of a non-serpentine soil (i.e. *Glomus sp.* 1 dominated).

### Comparison of Field versus Common Garden

**Figure 2:**
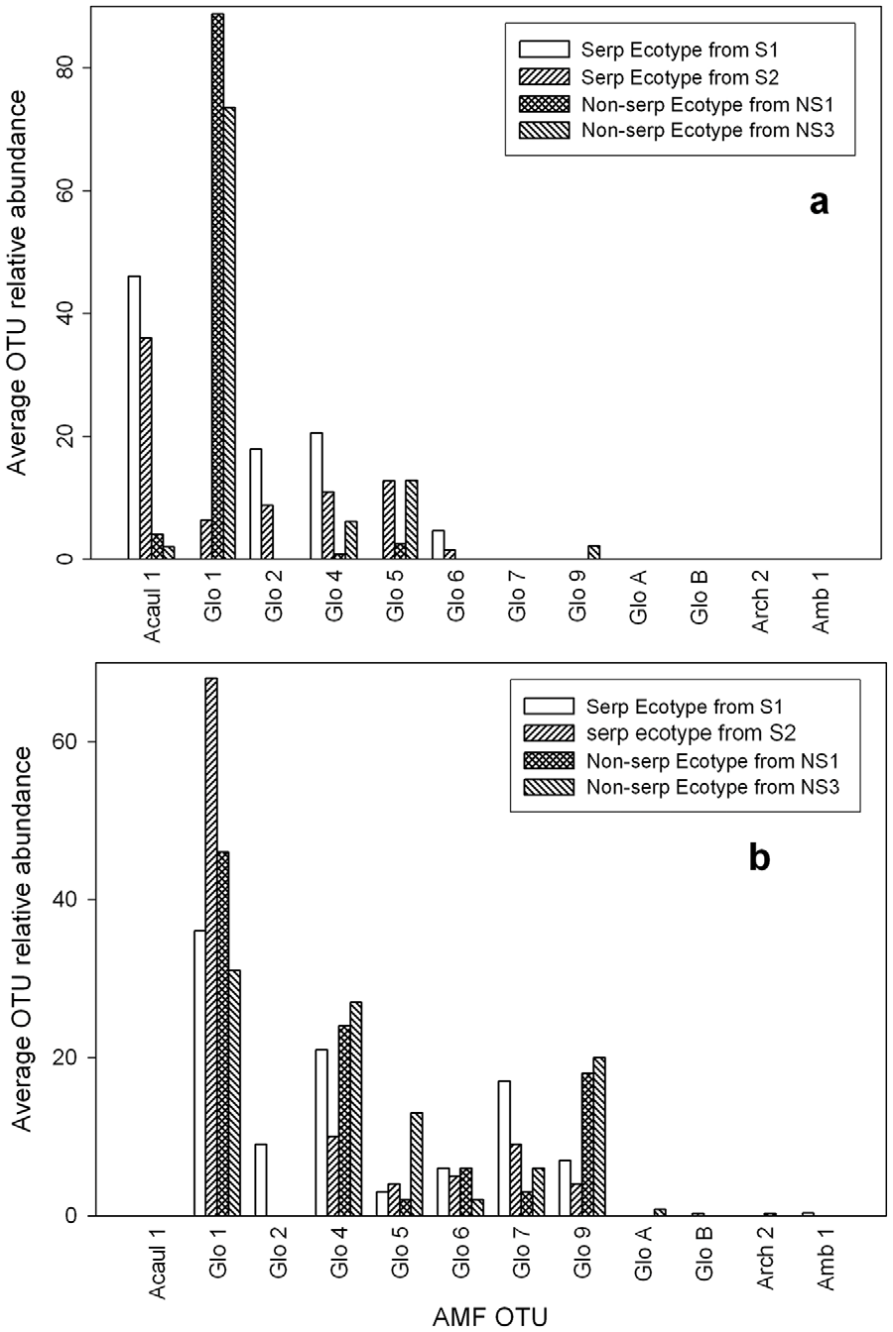
Comparison of the average OTU relative abundance of AMF associated with the *Collinsia sparsiflora* ecotype populations in the field versus in a common garden. *C. sparsiflora* ecotypes populations (S1, S2, NS1, and NS3) were grown in a) the field (Schechter and Bruns (2008) or grown in b) a common garden of mixed serpentine and non-serpentine AMF in the greenhouse.

We hypothesized that host-symbiont specificity would be indicated by a similar pattern of associated AMF taxa when grown in the common garden as found in the field. Comparing the average OTU relative abundance of AMF associated with the *C. sparsiflora* ecotype populations when grown in common garden with those when growing in the field shows a clear change in AMF OTU dominance (Figure 2). In the field, *Acaulospora sp.* 1 was the dominant OTU associated with the serpentine ecotypes populations (S1 and S2), while *Glomus sp.* 1 was the dominant OTU associated with the non-serpentine ecotype populations (NS1 and NS3) (Figure 2a). However, in the common garden experiment, *Glomus sp.* 1 now dominated both serpentine and non-serpentine ecotype populations, while *Acaulospora sp.* 1 was completely absent (Figure 2b). Thus, the common garden AMF assemblages showed a *Glomus sp.* 1 dominant assemblage pattern similar to the non-serpentine soil sampled in the field experiment. This is supported by ANOSIM and MDS data, which showed similarity between the common garden and non-serpentine-only control AMF assemblages and distinction from the serpentine-only control AMF assemblages (Figure 1).

The MDS ordination comparing soil nutrient concentrations between growth medium sampled from the common garden experiment and those found in serpentine and non-serpentine soil sampled from the field [19] clearly show that the common garden soil is clustered within the non-serpentine soils (Figure S3). The similarity between the common garden soil and non-serpentine soil is also clear when comparing individual nutrients (Table S1). Serpentine soils at McLaughlin Reserve are defined by having a Ca∶Mg<1 and non-serpentine soils as having Ca∶Mg>1 [6]. The common garden soil Ca∶Mg was 1.71 placing it in the “non-serpentine” category. However, this soil type is clearly a chimeric “non-serpentine” that has attributes distinct from the NS1 and NS3 non-serpentine soils used in the common garden.

## Discussion

### No host genotype – AM fungal genotype specificity

The primary goal of this experiment was to test if the pattern of specificity between adapted host ecotypes and AMF taxa found in the field was due to genotypic specificity between host ecotypes and adapted AMF taxa. We hypothesized that host-symbiont specificity would be indicated if, when grown in a common environment, *C. sparsiflora* serpentine ecotypes associated with a *Acaulospora sp.* 1 dominated AMF assemblage and non-serpentine ecotypes associated with a *Glomus sp.* 1 dominated AMF assemblage. Associating with the same AMF taxa when in a common environment as was found in the field would imply a genotype level affinity between adapted host and adapted AMF taxa, independent of soil edaphic conditions. Instead, we found that both serpentine and non-serpentine ecotypes associated with a similar set of AMF fungi (Figure 1) that was dominated by *Glomus sp.* 1 (Table 2). Consequently we have to reject host-symbiont specificity as the basis for distinction in serpentine and non-serpentine ecotype AMF assemblages in the field.

Host-symbiont specificity between adapted host and AMF taxa would have been an exceptional finding. True specificity between host-AM fungi has only been found in myco-heterotrophic plants [32], [33]. Only Helgason *et al.* [18] has demonstrated “selectivity” between photosynthetic host and a specific AM fungus across field soil and greenhouse medium. In our study, despite ecological evidence of adapted ecotype-AM fungal specificity, both host ecotypes behaved the same with respect to AMF when in a common soil. This result indicates soil type as a key factor in the community assembly of host-AMF associations in this serpentine/non-serpentine system. Other studies have shown that resource limitation [34], plant neighborhood [35], host priority effects [36], positive feedback [37], negative feedback [38], as well as host and symbiont adaptive responses [12], [13] all affect community assembly of host-AMF associations and response to the symbiosis. Thus evolutionary and ecological trajectories of plant-AMF interactions may be strongly context dependent.

While our results did not support host-symbiont specificity, ecotype AMF associations were not “random” as we proposed for the alternative hypothesis. In fact, based on differences in relative abundance of individual AMF taxa associates, serpentine ecotype populations S1 and S2 showed preference for distinct AMF taxa within the *Glomus sp.* 1 dominated AMF assemblage (Table 2). Plant-fungi preference has been seen at the host genotypic scale [17], [39], [40]. However, unlike many of those studies, we found no difference in growth response associated with “preference” for particular AMF assemblages (Table 2). The lack of association between growth response and differences in AMF assemblage may be due to the fact that all ecotypes were associating with a *Glomus sp.* 1 dominated assemblage (Table 2) and growing in a chimeric “non-serpentine” soil type (Figure S3).

To our knowledge, this study is the first AMF “common garden” test of host-AM fungal specificity. Knowledge of environmentally diverse plant ecotypes and AMF assemblages as well as the ability to identify root associated AMF taxa through molecular methods made this possible. However, our use of soil inoculum limited our ability to test for host ecotype-symbiont specificity in a truly “neutral” environment [41]. The chimeric soil of the common garden was not identical to any of the source soils and thus cannot be strictly considered “home” soil for any of the AMF taxa present [34], [42], [43], but we showed that the *C. sparsiflora* ecotypes and AMF were essentially functioning within a “non-serpentine” environment in our common garden, perhaps biasing our result.

We believe that using soil inoculum was the only way to ensure that all native serpentine and non-serpentine AMF found in the field had the chance to colonize *C. sparsiflora* ecotype populations in this experiment. Our previous attempts at producing single-species pot cultures of field AMF taxa for this experiment were unsuccessful. However, it is highly unlikely that all field AMF can be cultured under typical AMF culturing conditions (J. Morton, *pers. comm.*,). Ji *et al.* [42] got around this limitation in their AMF reciprocal transplant experiment by adding AMF spores extracted from field soils as the source of inoculum. In our case, repeated spore extractions from field soil yielded very few spores, indicating that soil mycelium and infected root pieces may be the primary source of AMF inoculum in our field conditions. Differences in inoculum potential between serpentine (S1 and S2) and non-serpentine (NS1 and NS3) soil inoculum could possibly account for the abundance of *Glomus sp.* 1 in the common garden mixture. However, our previous work [19] showed that serpentine and non-serpentine *C. sparsiflora* ecotypes had very similar levels of root colonization (the primary source of fungal inoculum in this system) and therefore likely similar inoculum potentials. Secondly, checking random root samples from serpentine and non-serpentine ecotypes collected for the common garden soil inoculum found equally high levels of AMF colonization in all ecotypes (>50%; pers. observ.), and since spores are not abundant in either soil, we should have provided equal inoculum from each source as root fragments. In addition, we also equalized the volume of roots from each ecotype sample added to the common garden. Thus, while differences in soil inoculum potential is possible we think it is unlikely a significant cause of AMF assemblage patterns found in this experiment.

Evidence from the common garden soil chemistry data and serpentine and non-serpentine controls indicate that soil type played a role in our findings. As a result of mixing rhizosphere soil from different soil types, the common garden environment was functioning as a “non-serpentine” (Ca∶Mg>1) soil type (Table S2; Figure S3). The AMF taxon *Glomus sp.* 1 has consistently been the dominant taxon associated with *C. sparsiflora* in non-serpentine soil plant and nearly absence in *C. sparsiflora* grown in serpentine soil. The dominance of *Glomus sp.* 1 in both serpentine and non-serpentine *C. sparsiflora* ecotypes grown in the common garden and non-serpentine control, its near absence in both ecotypes grown in the serpentine control and lack of evidence for dispersal limitation in the field suggests that the soil environment influenced the hosts' AMF associates. This is supported by a broader field study of AMF community assemblage across a serpentine/non-serpentine mosaic landscape [44]. In this study *Glomus sp.* 1 was dominant in non-serpentine sites and excluded from serpentine sites regardless of host species [44]. Therefore we believe that the results of this common garden experiment indicate soil type as a major factor determining *C. sparsiflora* AMF assemblage structure, instead of host genotype.

### The Greenhouse Effect on AMF Assemblages

It is known that disturbance and artificial environmental conditions (light, temperature, moisture, and pot size) associated with manipulative greenhouse experiments may alter AMF assemblage composition from those found in the field [11], [45]. In the present study a likely example of this was found in the serpentine AMF assemblages. The field dominant *Acaulospora* 1 was only present as one sequence under greenhouse conditions. This may be due to its lack of competitive ability under greenhouse growth conditions where the fungi associate with only one small *C. sparsiflora*. In the field, *C. sparsiflora* typically grows in patches of several individuals along with other plant species, which could represent an interconnected mycorrhizal network [46] that could provide much more carbon to a fungal individual than an individual *C. sparsiflora* in a small pot could. However, we are comparing these serpentine-only AMF assemblages to field serpentine ecotype AMF assemblages collected in March 2005. Therefore, the near absence of *Acaulospora 1* may be due to changes in its abundance in the field over a years time rather than greenhouse conditions [47], [48]. Sykorova *et al.* [45] also found changes in AMF taxa abundance between field and greenhouse conditions, which they suggested were caused by fungal successional changes between r and K strategists over the course of the experiment (3 months versus 10 months). The short duration of our experiment (3 months) makes successional changes seem unlikely to us.

Conversely, the low abundance and “hit and miss” pattern of *Glomus* 1 in the serpentine AMF controls was similar to what was found in the field (Table 2) [19]. This probably indicates that *Glomus* 1 does not function as well in serpentine soil, as it seems unlikely due to its low abundance in the field, as it is widespread in adjacent non-serpentine soil [19] and in the experimental soil mixtures. Furthermore in the non-serpentine AMF control and the common garden, *Glomus 1* is a good colonizer under greenhouse conditions, so low abundance of *Glomus 1* propagules alone does not explain its low abundance in the serpentine-only control. In addition, data comparing adjacent serpentine and non-serpentine AMF in the same field site indicate that *Glomus 1* is intolerant of serpentine soil [44]. The greenhouse conditions seemed to favor increased abundance of the serpentine-only greenhouse dominant *Glomus* 4, which was the second most abundant AMF OTU associated with ecotypes growing in serpentine soil in the field [19].

The AMF composition and abundance seen in non-serpentine controls in this study and field sampled plants in our previous study [19] were similar, but again showed some greenhouse-specific differences. *Glomus* 1 was once again the dominant OTU associated with ecotypes growing in non-serpentine soil (Table 2) [19]. However, *Glomus* 4 showed an increase from 5% in the field to over 20% under greenhouse conditions, again indicating that greenhouse conditions alter assemblage structure. *Glomus 4* is the only AMF taxa found abundantly in all field sites as well as all greenhouse conditions. Sykorova *et al.* [45] explained a similar pattern of presence and abundance across field and greenhouse conditions as the behavior of generalist taxa. The most surprising addition to the AMF OTUs found in the non-serpentine-only control was *Glomus* 6. In the field, *Glomus* 6 was restricted to serpentine soil and thus dubbed a “serpentine-specific” OTU in the field [19], however, it was detected in the non-serpentine-only control albeit only associated with the S2 serpentine ecotype (Table 2). In contrast, *Glomus* 6 was found in the serpentine-only control with every ecotype (Table 2). Although *Glomus* 6 can no longer be considered “serpentine-specific”, this difference in pattern of presence between serpentine and non-serpentine controls still implies a “preference” for serpentine soil.

### Conclusion

The results of the common garden experiment confirm that the distinction between AMF assemblages associated with serpentine and non-serpentine *C. sparsiflora* ecotypes in the field [19] was not due to adapted host-symbiont specificity. This leaves the second scenario, in which the distinction is due to AMF assemblages shaped by edaphic factors as the most likely situation.

## Supporting Information

Figure S1 Consensus tree (50% majority rule) from Mr. Bayes analysis showing the phylogenetic relationship of the AMF sequences (18S rDNA) obtained from roots sampled from common garden experiment from two serpentine (CGS1 and CGS2) and two nonserpentine (CGNS1 and CGNS3) ecotype populations and from the serpentine-only control samples (CGC_S) as well as the non-serpentine only controls (CGC_NS) of *Collinsia sparsiflora*, in bold. Additional sequences from roots sampled from three serpentine (S1, S2, S3) and three nonserpentine (NS1, NS2, NS3) ecotype populations of *Collinsia sparsiflora* field experiment were included. Letters directly behind site designation refers to an individual sample; no letter means that the sequence is a representative from a 98% consensus of sequences found in multiple samples within that site. Grey blocks encompass groups of sequences that are 98% similar and designate experiment OTUs (in white by Genus affiliation). Other sequences are Genbank accessions of closely related BLAST matches as well as Glomeromycota voucher sequences. Letters behind Genbank accessions refer to origin of the sequence (S = spore, E = environmental). The values above the branches are Bayesian posterior probabilities (bold) followed by bootstrap values (100 replicates in Garli maximum likelihood analysis), only support greater than 50 is shown. *Olpidium brassica* was used as an out-group. Topology was similar between Bayesian and Garli analyses.
(TIF)

Figure S2 Rarefaction curve of the total number of sequences sampled from serpentine ecotype (S1, S2) and non-serpentine ecotype (NS, NS3) populations of *Collinsia Sparsiflora* grown in a common garden of serpentine and non-serpentine AMF. Rarefaction curves were produced by the EstimateS version 8.0 Mao Tau estimator.
(TIF)

Figure S3 Non-metric multi-dimensional scaling (MDS) ordination of soil nutrients (N, P, K, Mg, Ca, Mg∶Ca, Zn, Mn, and Fe) associated with serpentine (S) and non-serpentine (NS) ecotype field populations of *Collinsia sparsiflora*, and soil sampled from the common garden soil (CSCG). Soil nutrients included were chosen by the BIOENV routine.
(TIF)

Table S1 Soil chemical variables (S = serpentine, NS = non-serpentine, and CG = common garden soil). Values are means with standard deviation below in parentheses. Nitrogen (as NO_3_) phosphorus (P, Weak Bray), potassium (K), magnesium (Mg), calcium (Ca), zinc (Zn), iron (Fe), copper (Cu), and boron (B) are reported in parts per million (ppm). Cation exchange capacity (CEC) is reported as milliequivalents per 100 grams of soil. Highlighted numbers indicate Ca∶Mg ratio; serpentine soils have a ratio much less than one and non-serpentine soils have ratios greater than one. Different letters within a column indicate significant differences at P<0.05.
(DOC)
