# Supplementary figures and images for: A Common Garden Test of Host-Symbiont Specificity Supports a Dominant Role for Soil Type in Determining AMF Assemblage Structure in Collinsia sparsiflora
Source: PLoS One. 2013 Feb 5;8(2):e55507. doi: 10.1371/journal.pone.0055507 (PMC3564749; doi:10.1371/journal.pone.0055507)

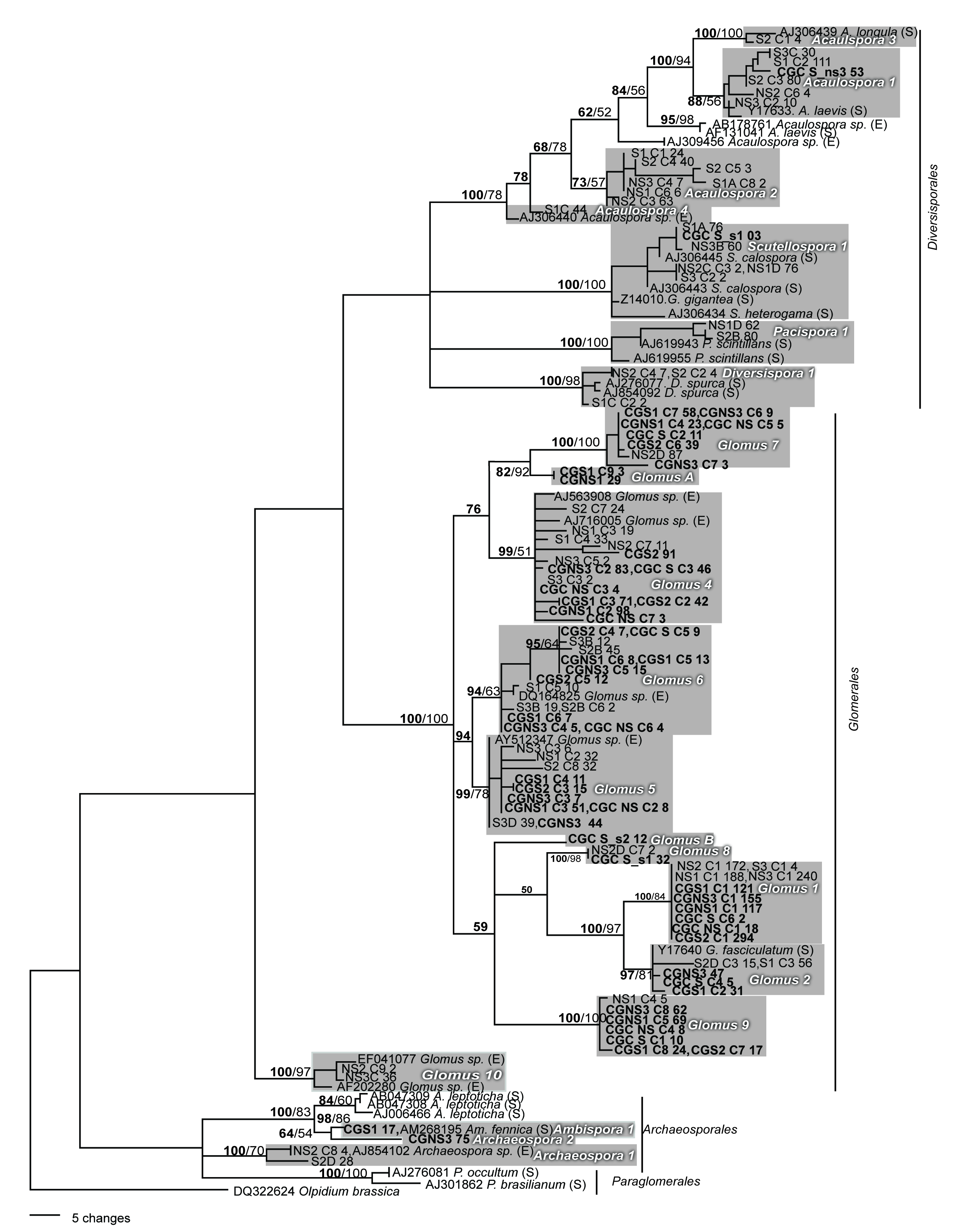

Supplement: Figure S1 — Consensus tree (50% majority rule) from Mr. Bayes analysis showing the phylogenetic relationship of the AMF sequences (18S rDNA) obtained from roots sampled from common garden experiment from two serpentine (CGS1 and CGS2) and two nonserpentine (CGNS1 and CGNS3) ecotype populations and from the serpentine-only control samples (CGC_S) as well as the non-serpentine only controls (CGC_NS) of Collinsia sparsiflora, in bold. Additional sequences from roots sampled from three serpentine (S1, S2, S3) and three nonserpentine (NS1, NS2, NS3) ecotype populations of Collinsia sparsiflora field experiment were included. Letters directly behind site designation refers to an individual sample; no letter means that the sequence is a representative from a 98% consensus of sequences found in multiple samples within that site. Grey blocks encompass groups of sequences that are 98% similar and designate experiment OTUs (in white by Genus affiliation). Other sequences are Genbank accessions of closely related BLAST matches as well as Glomeromycota voucher sequences. Letters behind Genbank accessions refer to origin of the sequence (S = spore, E = environmental). The values above the branches are Bayesian posterior probabilities (bold) followed by bootstrap values (100 replicates in Garli maximum likelihood analysis), only support greater than 50 is shown. Olpidium brassica was used as an out-group. Topology was similar between Bayesian and Garli analyses. (TIF) [file pone.0055507.s001.tif]

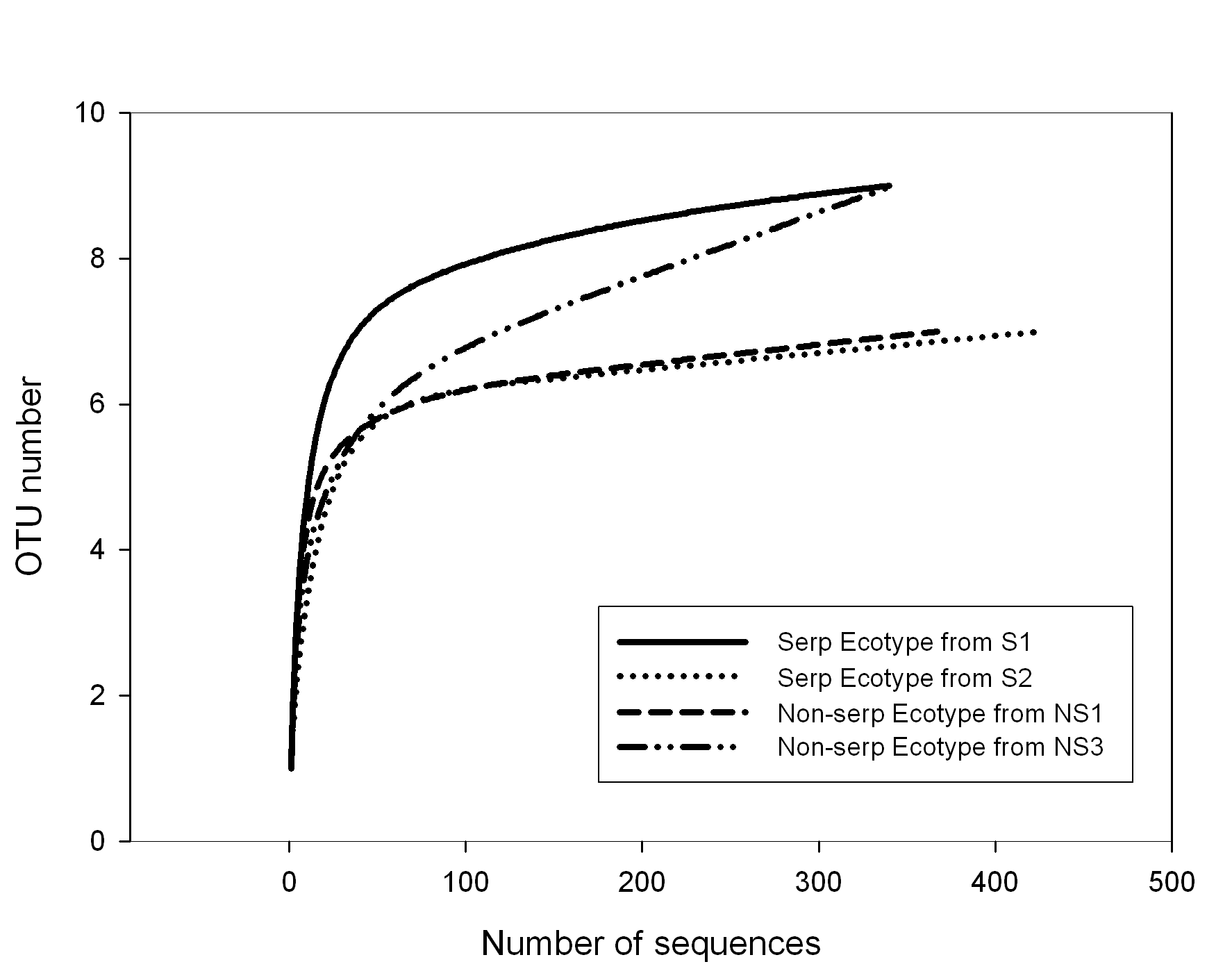

Supplement: Figure S2 — Rarefaction curve of the total number of sequences sampled from serpentine ecotype (S1, S2) and non-serpentine ecotype (NS, NS3) populations of Collinsia Sparsiflora grown in a common garden of serpentine and non-serpentine AMF. Rarefaction curves were produced by the EstimateS version 8.0 Mao Tau estimator. (TIF) [file pone.0055507.s002.tif]
