# Supplementary material for: A Common Garden Test of Host-Symbiont Specificity Supports a Dominant Role for Soil Type in Determining AMF Assemblage Structure in Collinsia sparsiflora
Source: PLoS One. 2013 Feb 5;8(2):e55507. doi: 10.1371/journal.pone.0055507 (PMC3564749; doi:10.1371/journal.pone.0055507)
Supplement: Table S1 — Soil chemical variables (S = serpentine, NS = non-serpentine, and CG = common garden soil). Values are means with standard deviation below in parentheses. Nitrogen (as NO3) phosphorus (P, Weak Bray), potassium (K), magnesium (Mg), calcium (Ca), zinc (Zn), iron (Fe), copper (Cu), and boron (B) are reported in parts per million (ppm). Cation exchange capacity (CEC) is reported as milliequivalents per 100 grams of soil. Highlighted numbers indicate Ca∶Mg ratio; serpentine soils have a ratio much less than one and non-serpentine soils have ratios greater than one. Different letters within a column indicate significant differences at P<0.05. (DOC) [file pone.0055507.s004.doc]

**Table S1**. Soil chemical variables (S = serpentine, NS = non-serpentine, and CG = common garden soil). Values are means with standard deviation below in parentheses. Nitrogen (as NO_3_) phosphorus (P, Weak Bray), potassium (K), magnesium (Mg), calcium (Ca), zinc (Zn), iron (Fe), copper (Cu), and boron (B) are reported in parts per million (ppm). Cation exchange capacity (CEC) is reported as milliequivalents per 100 grams of soil. Highlighted numbers indicate Ca:Mg ratio; serpentine soils have a ratio much less than one and non-serpentine soils have ratios greater than one. Different letters within a column indicate significant differences at P < 0.05.

| Site | N | P | K | Mg | Ca | Ca:Mg | Zn | Fe | pH |
| --- | --- | --- | --- | --- | --- | --- | --- | --- | --- |
| S1 | 2.50*b* | 3.75*c* | 111.75*b* | 1435.00*a* | 343.00*d* | 0.24 | 1.05*b* | 33.00*ab* | 7.00*a* |
|  | **(1.29)** | **(0.96)** | **(40.55)** | **(292.55)** | **(86.15)** | **(0.03)** | **(0.79)** | **(4.00)** | **(0.08)** |
| S2 | 1.50*b* | 2.00*c* | 65.75*b* | 1021.25*ab* | 292.50*d* | 0.30 | 0.65*b* | 22.25*c* | 6.98*a* |
|  | **(0.58)** | **(0.00)** | **(23.77)** | **(197.56)** | **(41.44)** | **(0.11)** | **(0.24)** | **(4.72)** | **(0.22)** |
| CG | 8.50*a* | 16.50*b* | 206.00*a* | 668.00*bc* | 1140.50*b* | 1.71 | 10.05*a* | 31.00*abc* | 6.55*b* |
|  | **(3.53)** | **(3.53)** | **(0.00)** | **(2.83 )** | **(17.68)** | **(0.04)** | **(1.34)** | **(1.50)** | **(0.07)** |
| NS1 | 2.50*b* | 16.00*b* | 278.25*a* | 860.75*b* | 2143.50*a* | 2.58 | 1.42*b* | 37.00*a* | 6.52*b* |
|  | **(0.58)** | **(4.16)** | **(28.89)** | **(162.60)** | **(162.60)** | **(0.69)** | **(0.19)** | **(4.69)** | **(0.05)** |
| NS2 | 7.25*a* | 7.00*c* | 246.50*a* | 801.25*b* | 869.50*c* | 1.18 | 0.98*b* | 35.50*ab* | 6.28*bc* |
|  | **(2.36)** | **(2.31)** | **(40.50)** | **(251.71)** | **(49.10)** | **(0.43)** | **(0.13)** | **(6.95)** | **(0.22)** |
| NS3 | 3.25*b* | 43.75*a* | 224.50*a* | 307.25*c* | 2287.25*a* | 7.57 | 0.75*b* | 26.25*bc* | 6.12*c* |
|  | **(1.89)** | **(5.32)** | **(19.94)** | **(48.08)** | **(47.42)** | **(1.06)** | **(0.06)** | **(2.63)** | **(0.10)** |
